# Galactomannan testing of bronchoalveolar lavage fluid is useful for diagnosis of invasive pulmonary aspergillosis in hematology patients

**DOI:** 10.1186/1471-2334-10-44

**Published:** 2010-03-03

**Authors:** Li-Yang Hsu, Ying Ding, Jason Phua, Liang-Piu Koh, Douglas S Chan, Kay-Leong Khoo, Paul A Tambyah

**Affiliations:** 1 Department of Medicine, National University Hospital, 5 Lower Kent Ridge Road, Singapore 119074, Singapore; 2 Division of Hematology, National University Cancer Institute, Singapore; 3 Department of Laboratory Medicine, National University Hospital, Singapore

## Abstract

**Background:**

Invasive pulmonary aspergillosis (IPA) is a major cause of morbidity and mortality in patients with hematological malignancies in the setting of profound neutropenia and/or hematopoietic stem cell transplantation. Early diagnosis and therapy has been shown to improve outcomes, but reaching a definitive diagnosis quickly can be problematic. Recently, galactomannan testing of bronchoalveolar lavage (BAL) fluid has been investigated as a diagnostic test for IPA, but widespread experience and consensus on optical density (OD) cut-offs remain lacking.

**Methods:**

We performed a prospective case-control study to determine an optimal BAL galactomannan OD cutoff for IPA in at-risk patients with hematological diagnoses. Cases were subjects with hematological diagnoses who met established definitions for proven or probable IPA. There were two control groups: subjects with hematological diagnoses who did not meet definitions for proven or probable IPA and subjects with non-hematological diagnoses who had no evidence of aspergillosis. Following bronchoscopy and BAL, galactomannan testing was performed using the Platelia *Aspergillus *seroassay in accordance with the manufacturer's instructions.

**Results:**

There were 10 cases and 52 controls. Cases had higher BAL fluid galactomannan OD indices (median 4.1, range 1.1-7.7) compared with controls (median 0.3, range 0.1-1.1). ROC analysis demonstrated an optimum OD index cutoff of 1.1, with high specificity (98.1%) and sensitivity (100%) for diagnosing IPA.

**Conclusions:**

Our results also support BAL galactomannan testing as a reasonably safe test with higher sensitivity compared to serum galactomannan testing in at-risk patients with hematological diseases. A higher OD cutoff is necessary to avoid over-diagnosis of IPA, and a standardized method of collection should be established before results can be compared between centers.

## Background

Invasive pulmonary aspergillosis (IPA) is a major cause of morbidity and mortality in immunocompromised patients, particularly those with hematological malignancies in the setting of profound neutropenia and/or hematopoietic stem cell transplantation [1]. Early diagnosis and therapy of IPA has been shown to improve outcomes [2], but reaching a definitive diagnosis quickly can be problematic in view of the lack of an exceptional diagnostic test, even with improved guidelines and newer test kits such as the Platelia *Aspergillus *seroassay (Bio-Rad Laboratories, Hercules, California, USA) and Fungitell 1,3 beta-D-glucan chromogenic assay (Associates of Cape Cod Inc., East Falmouth, Massachusetts, USA) [3,4].

Recently, investigators have explored the possibility of performing galactomannan testing on bronchoalveolar lavage (BAL) fluid samples from diverse patient populations [5-10]. Although invasive, BAL is associated with fewer complications than tissue biopsy, and BAL galactomannan testing appears to be more sensitive (60% - 100%) and specific (82% - 100%) than serum galactomannan testing for immunocompromised patients [5,6,8-10]. Two cutoff BAL galactomannan optical density (OD) indices have been proposed - ≥0.5 [5] and ≥1.0 [6-10] - although the amount of sterile saline instilled per BAL varied (40 ml to 150 ml) between studies, and few studies had well-defined negative controls.

## Methods

We attempted to determine an optimal BAL galactomannan OD cutoff for IPA in at-risk patients with hematological diagnoses in a prospective case-control study.

**Figure 1:**
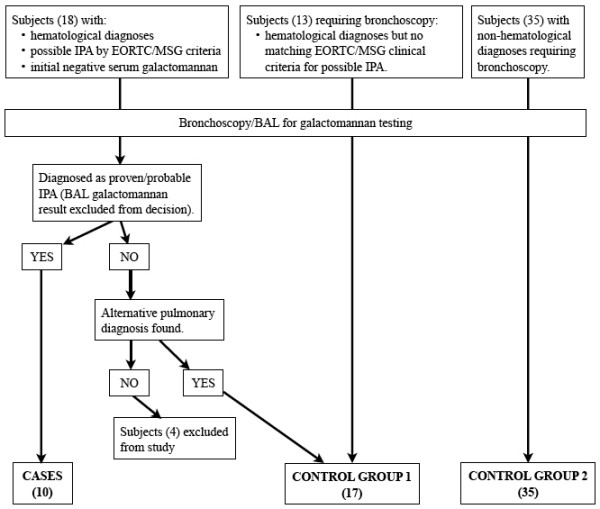
**Study schematic**.

All study subjects were recruited from our institute - a 997-bed tertiary acute-care hospital - between 1 September 2007 and 18 June 2009. The study schematic is shown in Figure 1. Cases were subjects with hematological diagnoses who matched the revised definitions for proven or probable IPA established by the European Organization for Research and Treatment of Cancer/Invasive Fungal Infections Cooperative Group (EORTC/MSG) [3], without the use of BAL galactomannan results. A single serum galactomannan OD cutoff of ≥ 0.7 was considered positive if retesting of the original sample gave the same result. At our institute, bronchoscopy for the diagnosis of lower respiratory tract invasive fungal disease (IFD) is performed only when a patient has host factors and clinical features as defined by EORTC/MSG [3], but the initial serum galactomannan testing using the Platelia *Aspergillus *seroassay is negative. Serum galactomannan testing is performed twice weekly for at-risk patients.

Controls were recruited from among other patients requiring bronchoscopy for diagnostic reasons. There were two control groups to determine if the BAL galactomannan test results differed among patients with or without haematological diagnoses:

1. Subjects with hematological diagnoses satisfying EORTC/MSG host factors criteria [3] who either:

a. Did not meet EORTC/MSG criteria for possible IFD OR

b. Met EORTC/MSG criteria for possible IFD but had an alternative confirmed pulmonary diagnosis other than IPA.

2. Subjects with non-hematological diagnoses who were not diagnosed with any form of aspergillosis.

Exclusion criteria included the use of piperacillin/tazobactam at the time of bronchoscopy as well as prolonged usage (more than 1 week) of any antifungal agent that was active against *Aspergillus *spp. prior to bronchoscopy.

BAL fluid samples were obtained by wedging the tip of the bronchoscope against the bronchus leading to the affected bronchopulmonary segment of maximal radiographic involvement, followed by instillation and aspiration of 40 to 60 ml aliquots of 0.9% sterile saline. Up to 10 ml of the first aliquot was sent for galactomannan testing. Other microscopy and microbiological tests were performed where ordered by the primary physicians. Transbronchial lung biopsies were performed where possible.

Galactomannan testing was performed on one sample of the BAL fluid per subject by investigators blinded to subject diagnoses and clinical characteristics. The Platelia *Aspergillus *seroassay was used in a HEPA-filtered biosafety cabinet according to the manufacturer's instructions. In brief, 300 μl of each BAL fluid sample was added to 100 μl of treatment solution, boiled for 3 minutes, and centrifuged at 10,000 g for 10 minutes. 50 μl each of supernatant and conjugate were mixed and incubated in microtiter plates pre-coated with monoclonal antibody EB-A2 for 90 min at 37°C. Wells were washed and incubated in the dark with 200 μl of chromogen solution for 30 minutes. Reactions were stopped and absorbance at 450 and 620 nm read using a plate-reader. Positive and negative controls (provided in the kit) were included in each assay. Results were recorded as an index relative to the OD of the cut-off control, and retesting of the BAL samples were performed if the OD index was ≥ 0.5. The optimal galactomannan OD index cutoff was determined by ROC analysis using STATA 10.1.

The institutional ethics committee approved this study and written informed consent was obtained from all subjects.

## Results

There were a total of 10 cases (1 proven and 9 probable IPA) and 52 controls (Figure 1). Four subjects with possible IFD but no definitive pulmonary diagnosis were excluded from the study after enrolment. Demographic, clinical and laboratory characteristics of cases and controls are shown in Table 1, while the relevant individual characteristics of cases are shown in Additional file 1: Table S1. Four cases had concomitant serum galactomannan OD index of ≥ 0.7 after initial negative tests, four cases had *Aspergillus *hyphae seen on cytology of BAL fluid - one also had positive BAL cultures for *Aspergillus fumigatus*, and three cases had acute-angle branching hyphae with tissue invasion seen on transbronchial lung biopsy. Nine cases had already received empiric anti-*Aspergillus *therapy prior to bronchoscopy, and their BAL fungal cultures were negative (Additional file 1: Table S1). Median time from the appearance of CT changes corresponding to EORTC criteria for IFD [3] to bronchoscopy was 3.5 days (range 1 to 6 days). This corresponded with the time between initiation of anti-*Aspergillus *therapy and bronchoscopy with the exception of the final case, where therapy was initiated after bronchoscopy.

**Table 1:** Demographic, clinical and laboratory characteristics of the study subjects.

	Cases (n = 10)	Control 1^a^ (n = 17)	Control 2^b^ (n = 35)
Median Age, years (Range)	35 (9 - 89)	43 (19 - 75)	63 (28 - 81)

Male gender (%)	8 (80.0)	13 (76.5)	24 (68.6)

Allogenic stem-cell transplantation (%)	2 (20.0)	8 (47.1)	0 (0.0)

Neutropenia (%) ^c^	9 (90.0)	11 (64.7)	0 (0.0)

Serum galactomannan testing:			
- Number tested (%)	10 (100.0)	11 (64.7)	1 (2.9)
- Median OD index (range)^d^	0.3 (0.1 - 1.8)	0.1 (0.0 - 0.2)	0.2

BAL galactomannan testing:			
- Median OD index (range)	4.1 (1.1 - 7.7)	0.2 (0.1 - 1.0)	0.4 (0.1 - 1.1)
- OD index ≥ 0.5 (%)	10 (100)	3 (17.6)	11 (31.4)

BAL fungal cultures (%):	10 (100)	17 (100)	18 (51.4)
- Candida spp. (%)	1 (10.0)	6 (35.3)	6 (33.3)
- Aspergillus spp. (%)	1 (10.0)	0 (0.0)	0 (0.0)

Primary pulmonary diagnoses:			
- IPA (%)	10 (100.0)	0 (0.0)	0 (0.0)
- Bacterial pneumonia (%)	0 (0.0)	4 (23.5)	14 (40.0)
- Lung cancer (%)	0 (0.0)	0 (0.0)	12 (34.3)
- Graft vs. host disease (%)	0 (0.0)	5 (29.4)	0 (0.0)
- Viral pneumonitis (%)	0 (0.0)	2 (11.8)	0 (0.0)
- Interstitial lung disease (%)	0 (0.0)	0 (0.0)	3 (8.6)
- Others (%)	0 (0.0)	6 (35.3)	6 (17.1)

^a ^Controls with underlying hematological diseases meeting EORTC/MSG host factors criteria for invasive fungal disease [3].

^b ^Controls with no underlying hematological diseases.

^c ^Absolute neutrophil count < 500/mm^3^

^d ^Highest serum galactomannan OD index within two weeks of bronchoscopy, if test was performed.

Four subjects in control group 1 met EORTC/MSG criteria for possible IPA with dense nodules visualized on CT thorax, but alternate pulmonary diagnoses were present for all - one had nocardiosis, one had *Mycobacterium abscesses *infection, one had mucormycosis, and the last had chronic pulmonary graft-versus-host disease. None of the four had BAL galactomannan OD indices exceeding 0.5, and three recovered without anti-*Aspergillus *therapy. The patient with mucormycosis died from subsequent gram-negative septicemia. CT features for the other subjects in this group comprised mainly pulmonary ground-glass infiltrates and/or consolidative changes. Only 11 (64.7%) subjects in control group 1 underwent serum galactomannan testing as the others were not considered at-risk for IFD.

Fourteen (26.9%) control subjects had BAL galactomannan OD indices that were ≥ 0.5. (Figure 1A) There was no common pulmonary diagnosis among them. BAL fungal culture was performed for 10 of these subjects, with *Candida *spp. isolated from seven samples. Thirteen did not receive anti-*Aspergillus *therapy and recovered uneventfully. The last was prescribed empiric caspofungin for persistent febrile neutropenia.

**Figure 2:**
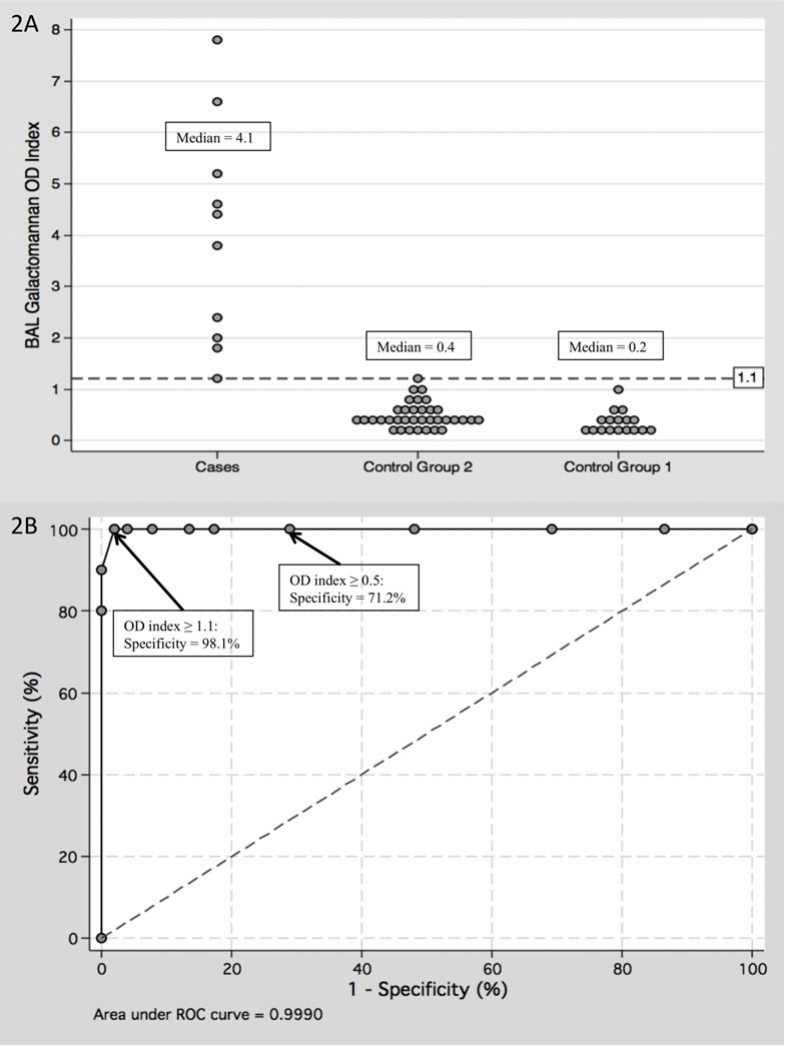
**Bronchoalveolar lavage fluid galactomannan optical density indices among cases and controls**. Figure 2A. Distribution of bronchoalveolar lavage (BAL) fluid galactomannan optical density (OD) indices among cases and controls. Figure 2B Receiver operating characteristic (ROC) analysis plot of BAL galactomannan OD indices among cases and controls.

Subjects in control group 2 tended to have higher BAL galactomannan OD indices (Figure 2A) compared to control group 1 subjects, but this was not statistically significant (*p *= 0.09). Combining the results of both groups resulted in a median BAL galactomannan OD index of 0.3 (range 0.1 - 1.1). In this case, the optimum OD index cutoff was ≥ 1.1 based on ROC analysis (Figure 2B), yielding a sensitivity and specificity of 100.0% (95%CI: 72.2% - 100%) and 98.1% (95%CI: 89.9% - 99.7%) respectively, and corresponding positive and negative predictive values of 90.9% and 100% respectively. Lowering the cutoff to ≥ 0.5 would result in significantly reduced specificity of 71.2% (95%CI: 59.8% - 83.2%), with corresponding positive and negative predictive values of 41.7% and 100% respectively.

No subject suffered significant adverse events from bronchoscopy.

## Discussion

Our study supports a higher galactomannan OD index cutoff (1.1) when using the Platelia *Aspergillus *seroassay on BAL fluid samples. This figure is higher than cutoffs proposed by others in certain well-defined patient populations [6-8,10], but the control subjects in our study are more heterogeneous, and may better represent a more diverse patient population. The results also support BAL galactomannan testing as a reasonably safe test with potentially higher sensitivity compared to serum galactomannan testing in at-risk patients with hematological diseases [6,7,10].

In the four cases with both positive serum and BAL galactomannan, the serum OD indices were lower than BAL OD indices, and time to positivity was longer for the former. This mirrored the results seen in an *in vitro *rabbit model of IPA, where the discordance was attributed to the time taken by advancing hyphae to breach the alveolar-capillary barrier [11].

False-positive BAL galactomannan tests have been reported for patients with airways colonized by either *Aspergillus *spp. or *Penicillium *spp. [8,9], but neither was cultured from the BAL fluid of our controls with high OD indices. Unlike the results of the Florida investigators, none of our non-immunocompromised subjects had OD indices exceeding 1.1, but our patient cohort was smaller [7].

One control had pulmonary mucormycosis and a low BAL galactomannan OD index. This particular case highlights the major deficiency of a galactomannan-based diagnostic algorithm: that the diagnosis of other emerging invasive moulds - albeit rare at present - may be delayed or missed.

There are several limitations to our study. Firstly, the number of subjects - particularly cases - is small, and this may affect the distribution of results. Secondly, bronchoscopy was only performed up to six days after radiological changes unlike in certain centers with immediate BALs [12]. Galactomannan testing is only performed twice weekly at our institute, and we believed it would be unethical to subject patients to the small but real risks of bronchoscopy if mycological evidence could be derived from serum galactomannan testing performed at the time when clinical features of IPA are present (there were three such patients during the study period). In early IPA, it is possible that the BAL galactomannan OD index may be lower than our proposed cutoff. Thirdly, OD measurements are dependent on the concentration of the galactomannan antigen, which in BAL fluid may be highly variable, being dependent on multiple factors including the amount of saline instilled during bronchoscopy, the area that is washed, time spent doing the lavage, and the amount of fluid retrieved. Our bronchoscopists instilled either 40 or 60 ml each time (all cases received 60 ml), and this difference may add variability to the results. The amount of sterile saline instilled varied significantly (40 to 150 ml) between studies [5-10], suggesting that the results - although similar - may not be comparable or extrapolated to other centers.

We are aware that standardizing BAL sampling is difficult especially given different clinical settings and patient sizes - 40 to 60 ml of saline instillation would be too much for a young child, but in an adult, occasionally there may be insufficient amount of fluid retrieved for testing. Nevertheless, standardization is important for inter-institutional comparisons, and for inclusion of BAL galactomannan testing OD cut-offs into international guidelines.

## Conclusions

In conclusion, our study demonstrates that BAL galactomannan testing is a safe and useful adjunct in the diagnosis of IPA. A higher cutoff for BAL as compared to serum galactomannan testing is necessary, however, to avoid over-diagnosis of IPA. A standardized method of collection should also be established before results can be comparable between centers. This should hopefully lead to better outcomes for our vulnerable patients.

## Competing interests

LYH has received research funding from Pfizer Inc. and Merck, Sharpe & Dohme. LPK is currently the site principal investigator for a multicenter study funded by Pfizer Inc. PAT has received research funding and honoraria from Pfizer Inc.

YD, JP, DSC and KLK report no potential conflicts of interest.

## Authors' contributions

LYH was the principal investigator and takes primary responsibility for the paper. YD and LPK recruited the patients and coordinated the research. JP and KLK performed and supervised the collection of specimens from the bronchoalveolar lavages. DSC performed the laboratory work for this study. LYH, JP and PAT wrote the paper.

All authors have read and approved the final manuscript.

## Pre-publication history

The pre-publication history for this paper can be accessed here:

http://www.biomedcentral.com/1471-2334/10/44/prepub

## Supplementary Material

Additional file 1 **Table S1 - Clinical characteristics of cases of proven/probable invasive pulmonary aspergillosis**. Clinical characteristics of individual cases of proven/probable invasive pulmonary aspergillosis. Click here for file
