# Supplementary material for: Galactomannan testing of bronchoalveolar lavage fluid is useful for diagnosis of invasive pulmonary aspergillosis in hematology patients
Source: BMC Infect Dis. 2010 Mar 3;10:44. doi: 10.1186/1471-2334-10-44 (PMC2837869; doi:10.1186/1471-2334-10-44)
Supplement: Additional file 1 — Table S1 - Clinical characteristics of cases of proven/probable invasive pulmonary aspergillosis. Clinical characteristics of individual cases of proven/probable invasive pulmonary aspergillosis. [file 1471-2334-10-44-S1.DOC]

## Table S1 - Clinical characteristics of cases of proven/probable invasive pulmonary aspergillosis.

| Case | Age | Gender | Hematological Diagnosis | BAL^a^ GM^b^ Index | Antifungal duration prior to BAL (days) | Serum GM^b^ Index^c^ | BAL^a^ Fungal Culture | BAL^a^ Cytology | Lung Biopsy (Histology) |
| --- | --- | --- | --- | --- | --- | --- | --- | --- | --- |
| 1 | 9 | Male | Acute myeloid leukemia | 2.4 | 6 | 0.9 | No growth | Negative | Not performed |
| 2 | 33 | Male | Acute lymphoblastic leukemia | 1.1 | 5 | 0.1 | No growth | Negative | Invasive hyphae seen |
| 3 | 89 | Male | Lymphoplasmacytoid lymphoma | 1.8 | 3 | 0.2 | No growth | Fungal hyphae seen | Not performed |
| 4 | 57 | Male | Acute myeloid leukemia | 2.0 | 4 | 0.7 | No growth | Negative | Not performed |
| 5 | 20 | Female | Acute lymphoblastic leukemia (allogenic bone marrow transplant) | 3.7 | 2 | 0.2 | No growth | Negative | Invasive hyphae seen |
| 6 | 20 | Male | Acute lymphoblastic leukemia | 4.4 | 4 | 1.8 | No growth | Negative | Invasive hyphae seen |
| 7 | 56 | Male | Acute myeloid leukemia | 4.5 | 3 | 0.3 | No growth | Fungal hyphae seen | Not performed |
| 8 | 54 | Male | T-cell angioimmunoblastic leukemia (allogenic bone marrow transplant) | 5.2 | 4 | 0.9 | No growth | Negative | Not performed |
| 9 | 37 | Male | Acute myeloid leukemia | 6.5 | 3 | 0.2 | No growth | Fungal hyphae seen | Not performed |
| 10 | 18 | Female | Hemophagocytic lymphohistiocytosis | 7.7 | 0 | 0.3 | *Aspergillus fumigatus* | Fungal hyphae seen | Not performed |

^a^ BAL = bronchoalveolar lavage

^b^ GM = galactomannan optical density

^c^ Highest serum galactomannan optical density index measurement within the episode of invasive pulmonary aspergillosis
